# Six new species of the spider genus *Spiricoelotes* species (Araneae, Agelenidae) from caves in Jiangxi, China

**DOI:** 10.3897/zookeys.561.6965

**Published:** 2016-02-08

**Authors:** Lu Chen, Zhe Zhao, Shuqiang Li

**Affiliations:** 1 Institute of Zoology, Chinese Academy of Sciences, Beijing 100101, China; 2 College of Life Sciences, Hebei University, Baoding, Hebei 071002, China

**Keywords:** Taxonomy, Asia, Coelotinae, description, diagnosis, cave

## Abstract

Six new species of the spider genus *Spiricoelotes* Wang, 2002 are described, *Spiricoelotes anshiensis* Chen & Li, **sp. n.** (♂♀), *Spiricoelotes chufengensis* Chen & Li, **sp. n.** (♂♀), *Spiricoelotes nansheensis* Chen & Li, **sp. n.** (♂♀), *Spiricoelotes taipingensis* Chen & Li, **sp. n.** (♂♀), *Spiricoelotes xianheensis* Chen & Li, **sp. n.** (♂♀) and *Spiricoelotes xiongxinensis* Chen & Li, **sp. n.** (♀). All new species were collected from caves in Jiangxi Province, China.

## Introduction

The spider genus *Spiricoelotes* was established by Wang (2002) for one coelotine species from China: *Coelotes zonatus* Peng & Wang, 1997. Three valid *Spiricoelotes* species were known before the current study: *Spiricoelotes urumensis* (Shimojana, 1989) from the Ryukyu Islands, *Spiricoelotes zonatus* (Peng & Wang, 1997) and *Spiricoelotes pseudozonatus* Wang, 2003 from China (World Spider Catalog 2015). In this paper, six new *Spiricoelotes* species are described. All new species were collected from caves in the Jiangxi Province of China.

## Material and methods

Specimens were examined with a LEICA M205C stereomicroscope. Images were captured with an Olympus C7070 wide zoom digital camera (7.1 megapixels) mounted on an Olympus SZX12 dissecting microscope. Epigynes and male palps were examined after dissection from the spiders’ bodies.

All measurements were obtained using a LEICA M205C stereomicroscope and are given in millimeters. Leg measurements are shown as: total length (femur, patella + tibia, metatarsus, tarsus). Only structures (palp and legs) on the left side of the body were described and measured. The abbreviations and terminology used in the text follows Wang (2002). Abbreviations used in this paper and in the figure legends: A = epigynal atrium; ALE = anterior lateral eye; AME = anterior median eye; AME-ALE = distance between AME and ALE; AME-AME = distance between AME and AME; ALE-PLE = distance between ALE and PLE; CD = copulatory duct; CDA = dorsal conductor apophysis; CF = cymbial furrow; E = embolus; EB = embolic base; ET = epigynal teeth; FD = fertilization duct; H = epigynal hood; LTA = dorso-retrolateral tibial apophysis; OC = outgrowth of conductor; PA = patellar apophysis; PLE = posterior lateral eye; PME = posterior median eye; PME-PLE = distance between PME and PLE; PME-PME = distance between PME and PME; RTA = retrolateral tibial apophysis; S = spermatheca; SH = spermathecal head; SST = spermathecal stalk; ST = subtegulum; T = tegulum.

A partial fragment of the mitochondrial gene cytochrome oxidase subunit I (COI) was amplified and sequenced for *Spiricoelotes anshiensis* sp. n., *Spiricoelotes chufengensis* sp. n., *Spiricoelotes nansheensis* sp. n., *Spiricoelotes taipingensis* sp. n., *Spiricoelotes xianheensis* sp. n. and *Spiricoelotes xiongxinensis* sp. n. following the protocol in Miller et al. (2009). Primers used in this study are: LCO1490 (5’-CWACAAAYCATARRGATATTGG-3’) (Folmer et al. 1994) and HCO2198zz (5’-TAAACTTCCAGGTGACCAAAAAATCA-3’) (this study). All sequences were blasted in GenBank and the accession numbers are provided in Table 1.

**Table 1.** Voucher specimen information.

Species	GenBank accession number	Sequence length	Collection localities
*Spiricoelotes anshiensis* sp. n.	KT896546	1196 bp	Chayuan Village, Shacun Town, Ji’an City, Jiangxi Province, China
*Spiricoelotes chufengensis* sp. n.	KT896541	1205 bp	Huangguan Village, Ningdu County, Ganzhou City, Jiangxi Province, China
*Spiricoelotes nansheensis* sp. n.	KT896544	1232 bp	Shuangqiao Town, Wanzai County, Yichun City, Jiangxi Province, China
*Spiricoelotes taipingensis* sp. n.	KT896542	1029 bp	Huangguan Village, Ningdu County, Ganzhou City, Jiangxi Province, China
*Spiricoelotes xianheensis* sp. n.	KT896543	1208 bp	Niedu Village, Chongyi County, Ganzhou City, Jiangxi Province, China
*Spiricoelotes xiongxinensis* sp. n.	KT896545	1232 bp	Zhanshan Village, Shangli County, Pingxiang City, Jiangxi Province, China

All specimens (including molecular vouchers) are deposited in the Institute of Zoology, Chinese Academy of Sciences in Beijing (IZCAS).

## Taxonomy

### Family Agelenidae C.L. Koch, 1837 Subfamily Coelotinae F.O.P.-Cambridge, 1893

#### Genus Spiricoelotes

Taxon classificationAnimaliaAraneaeAgelenidae

Wang, 2002

Spiricoelotes Wang, 2002: 129. Type species *Coelotes zonatus* Peng & Wang, 1997, from China.

##### Diagnosis.

**Figure 1.**
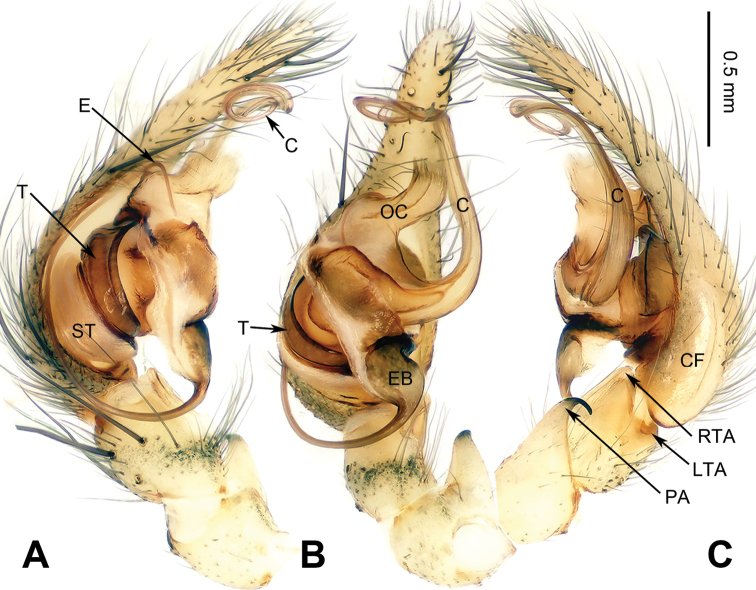
Left palp of *Spiricoelotes anshiensis* sp. n., holotype. **A** Prolateral **B** Ventral **C** Retrolateral. Scale bar equal for **A, B** and **C**.

**Figure 2.**
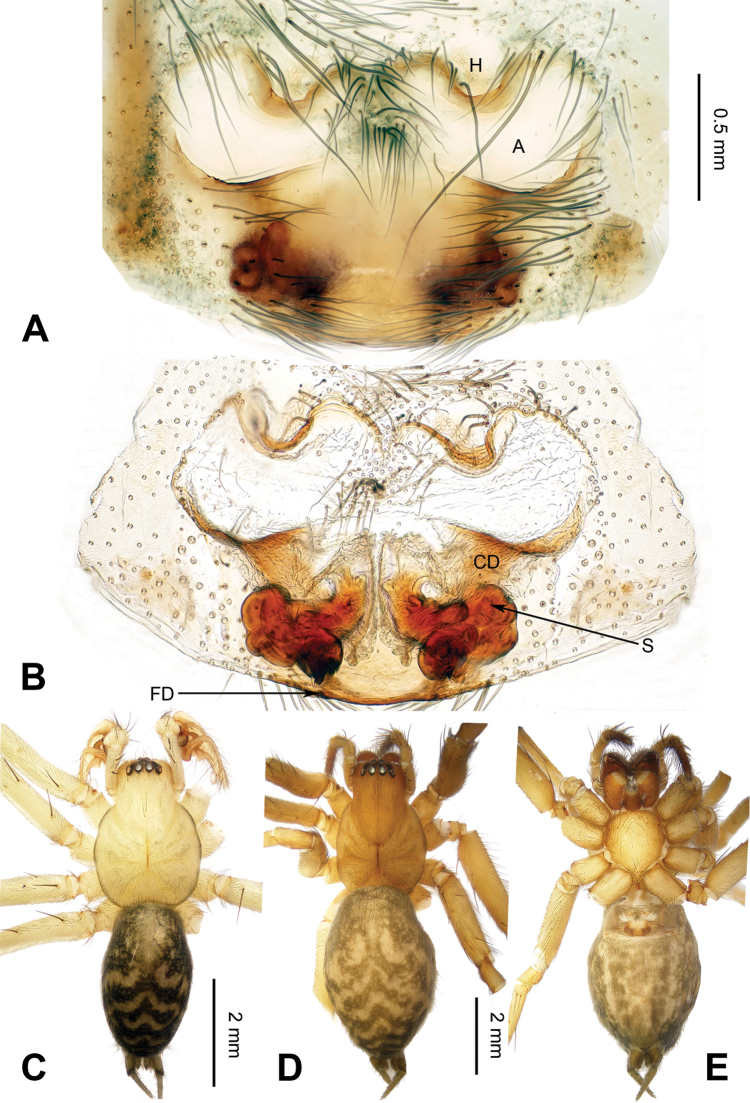
Epigyne and habitus of *Spiricoelotes anshiensis* sp. n., **A** Epigyne, ventral **B** Vulva, dorsal **C** Male habitus, dorsal **D** Female habitus, dorsal **E** Female habitus, ventral. Scale bars equal for **A** and **B**, equal for **D** and **E**.

The males can be easily recognized from other coelotines by the strongly curved patellar apophyses, the absence of a dorsal apophysis of the conductor, and the slender, anteriorly extending conductor. (Fig. 1A–C). The females can be distinguished from other coelotines by the absence of epigynal teeth, the well-developed epigynal hoods and the long, strongly convoluted spermathecae (Fig. 2A–B).

#### Spiricoelotes anshiensis

Taxon classificationAnimaliaAraneaeAgelenidae

Chen & Li sp. n.

http://zoobank.org/50A54C0B-F2F3-4663-AFC2-A8923FA64660

Figs 1 , 2 , 12

##### Type material.

**Holotype** ♂: China: Jiangxi: Ji’an City: Shacun Town, Chayuan Village, Anshi Cave, N26°31'24", E115°06'48", elevation: 332 m, 3.V.2013, Y.F. Luo and J.C. Liu. **Paratypes**: 5♀, same data as holotype.

##### Etymology.

The specific name refers to the type locality; adjective.

##### Diagnosis.

The male of the new species can be easily distinguished from all other *Spiricoelotes* by the long conductor (more than 1/2 length of cymbium, in previously known species subequal to or less than 1/2 length of cymbium) and looped conductor apex (with one loop, conductor of previously known species spiralled, rather than with looped apex), the long and broad outgrowth of the conductor (conductor of previously known species without outgrowth) (Fig. 1A–C). The female of the new species can be easily distinguished from all of the other *Spiricoelotes* by the bean-shaped atria (approximately 1/3 of epigynal plate, atria of previously known species obviously less than 1/3 of epigynal plate), the funnel-shaped copulatory ducts, the short spermathecae and the epigynal hoods close to each other (Fig. 2A–B).

##### Description.

**Male (holotype)**: Total length 5.59. Carapace 2.78 long, 1.94 wide. Abdomen 2.81 long, 1.63 wide. Eye sizes and interdistances: AME 0.14, ALE 0.15, PME 0.13, PLE 0.11; AME-AME 0.04, AME-ALE 0.02, PME-PME 0.06, PME-PLE 0.08. Leg measurements: I 11.50 (3.35, 3.45, 2.75, 1.95); II 12.24 (3.20, 4.16, 3.12, 1.76); III 10.84 (2.88 3.28, 2.88, 1.80); IV 14.57 (3.92, 4.10, 4.23, 2.32). Chelicerae with 3 promarginal and 4 retromarginal teeth. Palp: patellar apophysis longer than patella, with pointed tip, strongly curved; RTA with pointed tip, extending beyond the tibia; LTA short, approximately 1/4 length of RTA; cymbial furrow long, approximately 1/3 length of cymbium; conductor long, slender, anteriorly extending, and apex looped; with outgrowth, located at the base of conductor; embolus beginning at 5:30 to 6 o’clock position (Fig. 1A–C).

**Female (one of the paratypes)**: Total length 7.68. Carapace 3.28 long, 2.50 wide. Abdomen 4.40 long, 3.00 wide. Eye sizes and interdistances: AME 0.17, ALE 0.25, PME 0.19, PLE 0.17; AME-AME 0.04, AME-ALE 0.04, PME-PME 0.09, PME-PLE 0.08. Leg measurements: I 13.21 (3.60, 4.45, 3.12, 2.04); II 11.82 (3.50, 3.96, 2.60, 1.76); III 10.68 (3.04, 3.28, 2.76, 1.60); IV 14.88 (3.96, 4.85, 3.95, 2.12). Chelicerae as in male. Epigyne: atria bean-shaped, approximately 1/3 of epigynal plate, situated anteriorly and separated by septum (narrower than atria); hoods distinct, situated anteriorly, close to each other; spermathecae long and convoluted; copulatory ducts short, funnel-shaped (Fig. 2A–B).

##### Distribution.

Known only from the type locality (Fig. 12).

#### Spiricoelotes chufengensis

Taxon classificationAnimaliaAraneaeAgelenidae

Chen & Li sp. n.

http://zoobank.org/158012CD-B021-4A80-8A0C-F440F17E7D04

Figs 3 , 4 , 12

##### Type material.

**Holotype** ♂: China: Jiangxi: Ganzhou City: Ningdu County: Huangguan Village, Chufeng Cave, N26°29'35", E115°55'45", elevation: 395 m, 29.IV.2013, Y.F. Luo and J. Liu. **Paratypes**: 6♀, same data as holotype.

##### Etymology.

The specific name refers to the type locality; adjective.

##### Diagnosis.

**Figure 3.**
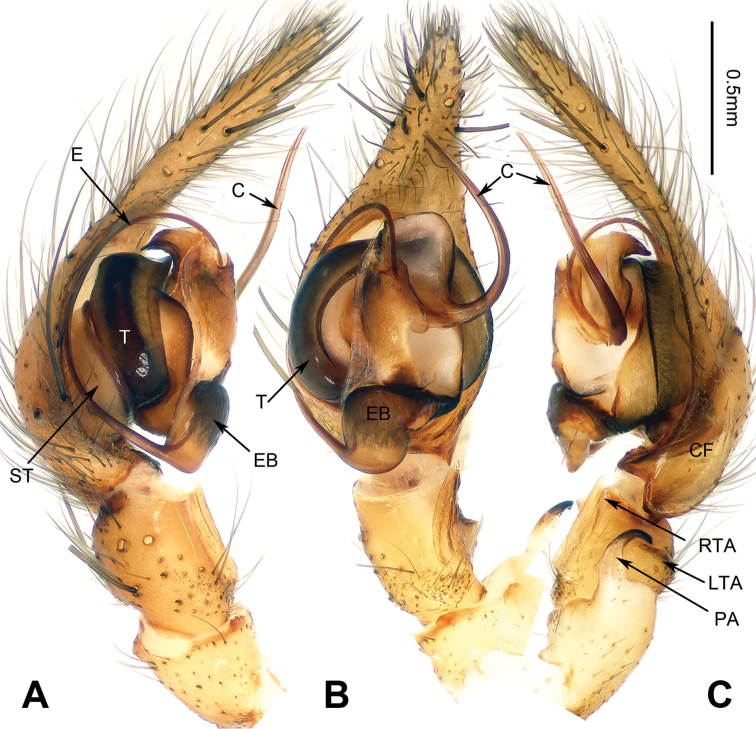
Left palp of *Spiricoelotes chufengensis* sp. n., holotype. **A** Prolateral **B** Ventral **C** Retrolateral. Scale bar equal for **A, B** and **C**.

**Figure 4.**
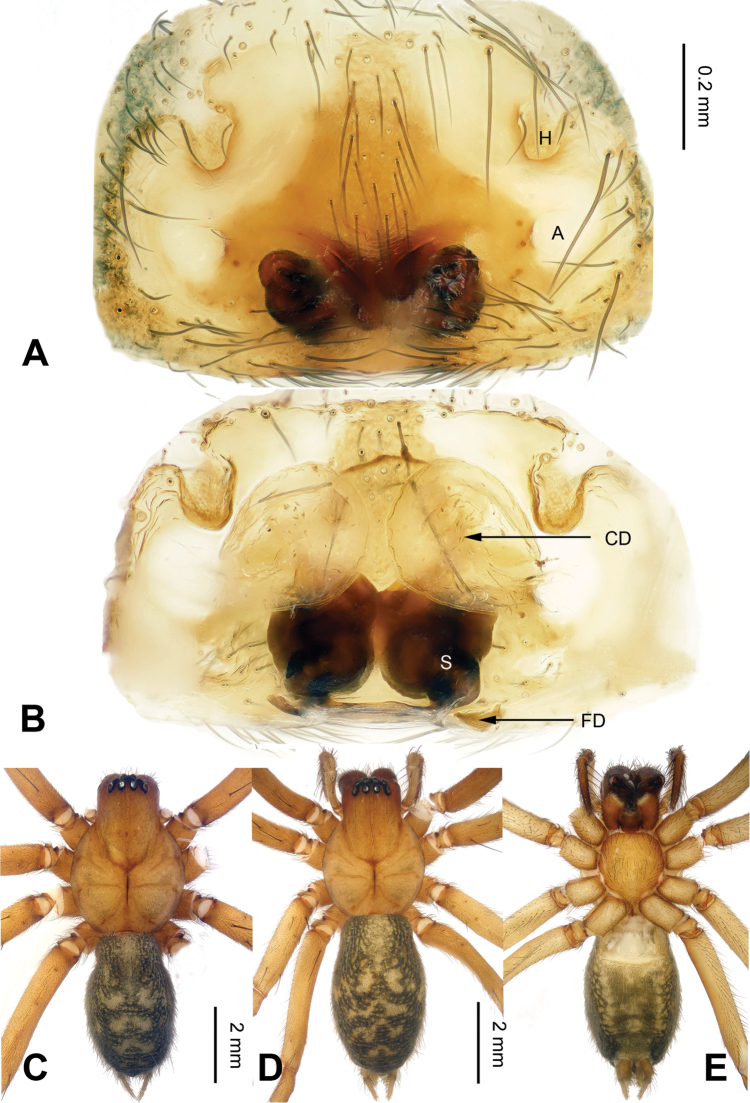
Epigyne and habitus of *Spiricoelotes chufengensis* sp. n., **A** Epigyne, ventral **B** Vulva, dorsal **C** Male habitus, dorsal **D** Female habitus, dorsal **E** Female habitus, ventral. Scale bars equal for **A** and **B**, equal for **D** and **E**.

The male of the new species has a uniquely shaped male palp and can be easily distinguished from all other *Spiricoelotes* by the more slender, needle-like conductor (conductor spiralled or with looped apex in other species) and the shorter cymbial furrow (approximately 1/4 length of cymbium, in other species approximately 1/2 length of cymbium) (Fig. 3A–C). The female of the new species can be easily distinguished from all of the other *Spiricoelotes* by the smaller (subequal to hoods) and posterolaterally situated epigynal atria, the bulb-shaped copulatory ducts and the broader spermathecae (Fig. 4A–B).

##### Description.

**Male (holotype)**: Total length 8.05. Carapace 4.25 long, 3.00 wide. Abdomen 3.80 long, 2.25 wide. Eye sizes and interdistances: AME 0.20, ALE 0.14, PME 0.18, PLE 0.18; AME-AME 0.08, AME-ALE 0.04, PME-PME 0.14, PME-PLE 0.13. Leg measurements: I 15.05 (4.23, 5.12, 3.45, 2.25); II 13.55 (3.85, 4.30, 3.35, 2.05); III 12.85 (3.75, 4.05, 3.25, 1.80); IV 16.80 (4.65, 4.95, 4.75, 2.45). Chelicerae with three promarginal and five retromarginal teeth. Palp: patellar apophysis long, subequal to width of patella, with pointed tip, strongly curved; RTA with pointed tip, extending slightly beyond the tibia; LTA short; cymbial furrow short, approximately 1/4 length of cymbium; conductor long, needle-like, anteriorly extending; embolus, beginning at 6 o’clock to 6:30 position (Fig. 3A–C).

**Female (one of the paratypes)**: Total length 7.52. Carapace 3.60 long, 2.48 wide. Abdomen 3.92 long, 2.36 wide. Eye sizes and interdistances: AME 0.15, ALE 0.19, PME 0.14, PLE 0.18; AME-AME 0.06, AME-ALE 0.03, PME-PME 0.11, PME-PLE 0.09. Leg measurements: I 13.15 (3.75, 4.45, 3.03, 1.92); II 11.24 (3.23, 3.80, 2.49, 1.72); III 10.62 (2.85, 3.32, 2.60, 1.85); IV 14.05 (3.80, 4.54 , 3.80, 2.00). Chelicerae as in male. Epigyne: atria small, located posterolaterally; hoods distinct, located anterolaterally; spermathecae broad, convoluted; copulatory ducts bulb-shaped (Fig. 4A–B).

##### Distribution.

Known only from the type locality (Fig. 12).

#### Spiricoelotes nansheensis

Taxon classificationAnimaliaAraneaeAgelenidae

Chen & Li sp. n.

http://zoobank.org/FD982663-7E2B-4493-A395-4A6E5FD4694E

Figs 5 , 6 , 12

##### Type material.

**Holotype** ♂: China: Jiangxi: Yichun City: Wanzai County: Shuangqiao Town, Nanshe Cave, N28°10'8", E114°17'16", elevation: 195 m, 15.V.2013, Y.F. Luo and J.C. Liu. **Paratypes**: 1♂10♀, same data as holotype.

##### Etymology.

The specific name refers to the type locality; adjective.

##### Diagnosis.

**Figure 5.**
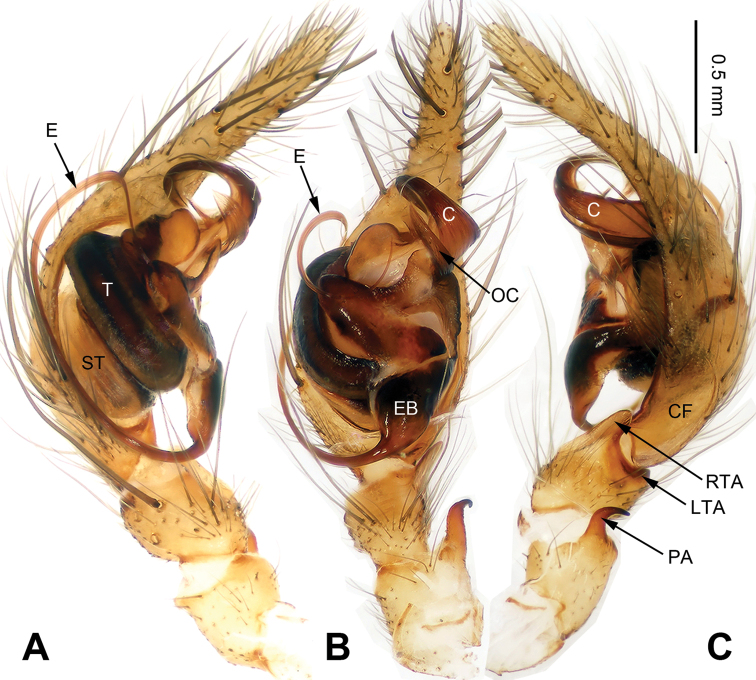
Left palp of *Spiricoelotes nansheensis* sp. n., holotype. **A** Prolateral **B** Ventral **C** Retrolateral. Scale bar equal for **A, B** and **C**.

**Figure 6.**
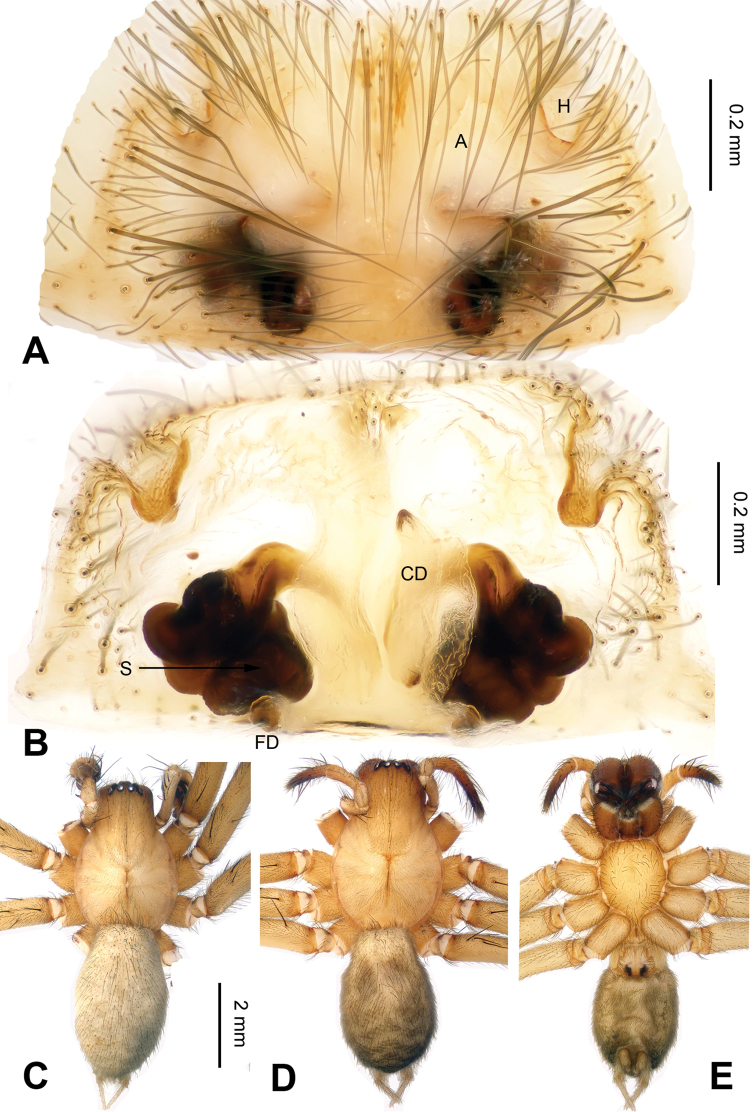
Epigyne and habitus of *Spiricoelotes nansheensis* sp. n., **A** Epigyne, ventral **B** Vulva, dorsal **C** Male habitus, dorsal **D** Female habitus, dorsal **E** Female habitus, ventral. Scale bars equal for **C, D** and **E**.

The male of the new species has uniquely shaped palps and can be easily recognized from all of the other *Spiricoelotes* species by the extremely modified conductor (with two outgrowths at the base, only one in *Spiricoelotes anshiensis* sp. n., and none in other species), the looped apex (Fig. 5A–C). The female of the new species is similar to *Spiricoelotes chufengensis* sp. n. but can be distinguished from it by the larger and anteriorly situated atria, the more slender and longer copulatory ducts, the longer, more slender spermathecae that are separated from each other by copulatory ducts (Fig. 6A–B).

##### Description.

**Male (holotype)**: Total length 7.00. Carapace 3.40 long, 2.36 wide. Abdomen 3.60 long, 2.16 wide. Eye sizes and interdistances: AME 0.13, ALE 0.16, PME 0.15, PLE 0.08; AME-AME 0.05, AME-ALE 0.04, PME-PME 0.09, PME-PLE 0.06. Leg measurements: I 14.05 (3.60, 4.60, 3.55, 2.30); II 13.06 (3.50, 4.00, 3.35, 2.21); III 12.30 (3.44, 3.56, 3.33, 1.97); IV 16.05 (4.20, 5.00, 4.85, 2.00). Chelicerae with three promarginal and four retromarginal teeth. Palp: patellar apophysis long, subequal to patellar width, with pointed tip, strongly curved; RTA with blunt tip, extending beyond the tibia; LTA long, approximately 1/3 length of RTA; cymbial furrow short, approximately 1/5 length of cymbium; conductor broad, with looped apex, with two outgrowths at the base; embolus originates at 6 o’clock position (Fig. 5A–C).

**Female (one of paratypes)**: Total length 7.16. Carapace 3.84 long, 2.48 wide. Abdomen 3.32 long, 2.08 wide. Eye sizes and interdistances: AME 0.10, ALE 0.15, PME 0.14, PLE 0.16; AME-AME 0.01, AME-ALE 0.04, PME-PME 0.09, PME-PLE 0.13. Leg measurements: I 13.80 (3.85, 4.10, 3.75, 2.10); II 13.08 (3.60, 4.08, 3.20, 2.20; III 12.68 (3.27, 3.96, 3.40, 2.05); IV 16.45 (4.15, 5.05, 4.80, 2.45); IV 16.66 (4.68, 5.00, 4.61, 2.37). Chelicerae as in male. Epigyne: atria small, located anteriorly, close to each other; hoods located anterolaterally; spermathecae long, convoluted; copulatory ducts slender, looped, located at center of vulva (Fig. 6A–B).

##### Distribution.

Known only from the type locality (Fig. 12).

#### Spiricoelotes taipingensis

Taxon classificationAnimaliaAraneaeAgelenidae

Chen & Li sp. n.

http://zoobank.org/D21CD9B1-63DC-4A86-9F9D-4DF0CBB8622F

Figs 7 , 8 , 12

##### Type material.

**Holotype** ♂: China: Jiangxi: Ganzhou City: Ningdu County: Huangguan Village, Taiping Cave, N25°28'53", E115°54'35", elevation: 420 m, 29.IV.2013, Y.F. Luo and J.C. Liu. **Paratypes**: 10♀, same data as holotype.

##### Etymology.

The specific name refers to the type locality; adjective.

##### Diagnosis.

**Figure 7.**
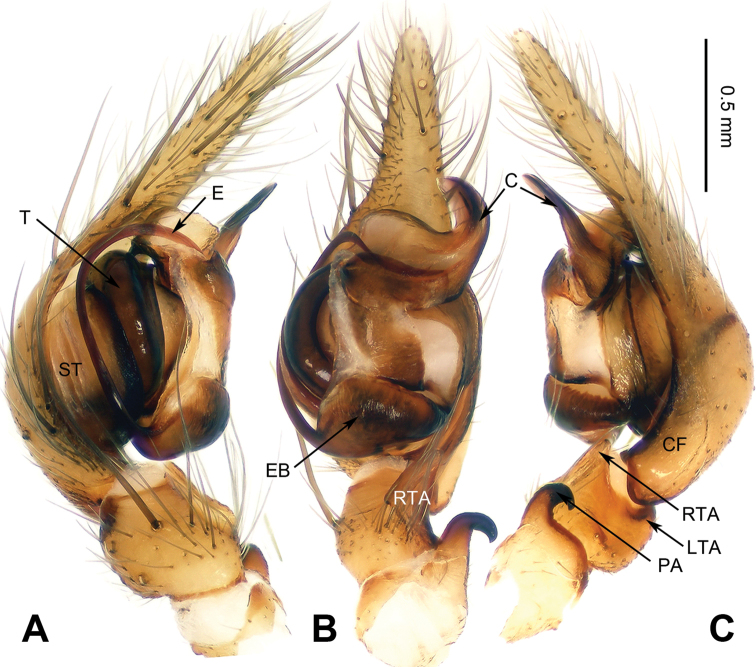
Left palp of *Spiricoelotes taipingensis* sp. n., holotype. **A** Prolateral **B** Ventral **C** Retrolateral. Scale bar equal for **A, B** and **C**.

**Figure 8.**
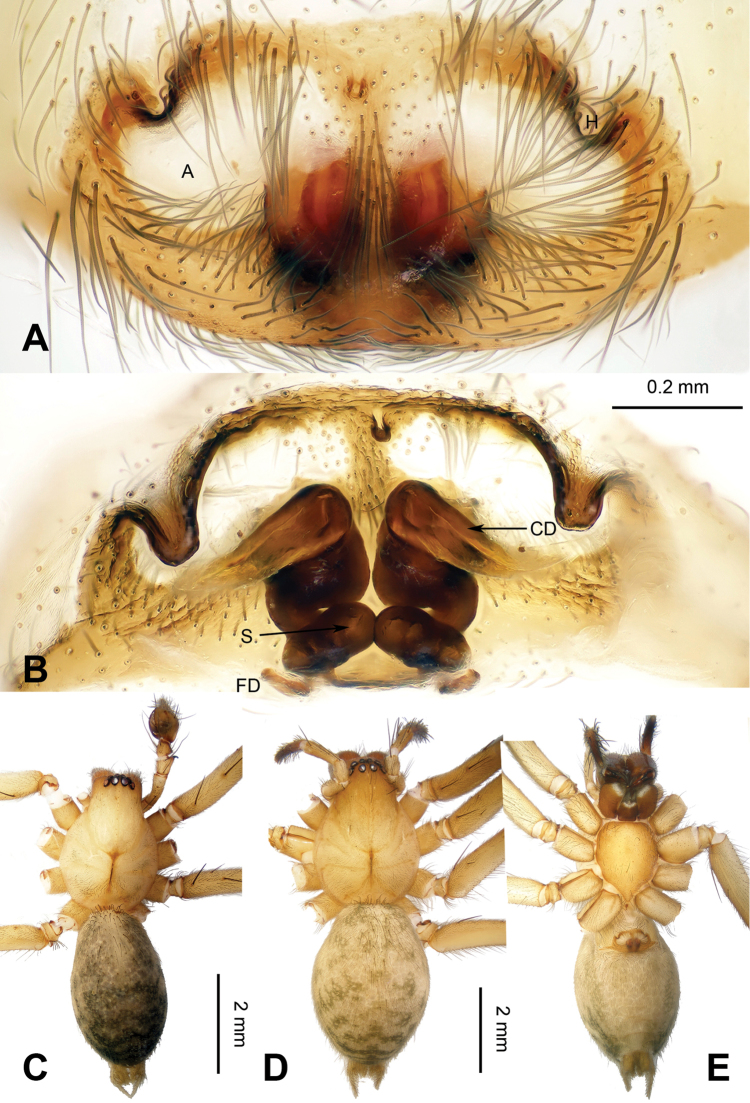
Epigyne and habitus of *Spiricoelotes taipingensis* sp. n., **A** Epigyne, ventral **B** Vulva, dorsal **C** Male habitus, dorsal **D** Female habitus, dorsal **E** Female habitus, ventral. Scale bars equal for **A** and **B**, equal for **D** and **E**.

The male of the new species can be easily recognized by the short, broad conductor without a looped apex, the short cymbial furrow (approximately 1/5 length of cymbium, approximately 1/2, 1/3 or 1/4 length of cymbium in other species) and the strong patellar apophysis (twice as broad as in other species) (Fig. 7A–C). The female of the new species is similar to *Spiricoelotes anshiensis* sp. n. but can be distinguished by the larger atria (approximately 1/2 of epigynal plate), the epigynal hoods that are separated by twice their width, the slender and horizontally stretched copulatory ducts, the longer and helical spermathecae (Fig. 8A–B).

##### Description.

**Male (holotype)**: Total length 5.80. Carapace 2.60 long, 1.68 wide. Abdomen 3.20 long, 2.00 wide. Eye sizes and interdistances: AME 0.13, ALE 0.15, PME 0.16, PLE 0.15; AME-AME 0.02, AME-ALE 0.02, PME-PME 0.06, PME-PLE 0.04. Leg measurements: I 14.81 (3.76, 4.81, 3.84, 2.40); II 12.10 (2.82, 3.80, 3.52, 1.96); IV 16.81 (4.25, 5.00, 5.06, 2.50). Chelicerae with three promarginal and four retromarginal teeth. Palp: patellar apophysis subequal to patellar width, strongly curved; RTA with blunt tip, extending slightly beyond distal margin of tibia; LTA long, about 1/2 length of RTA; cymbial furrow short, approximately 1/5 length of cymbium; conductor short, anteriorly extending; embolus beginning at 7 o’clock position; base of embolus wider than tibia, triangular, horizontally directed (Fig. 7A–C).

**Female (one of the paratypes)**: Total length 7.16. Carapace 3.36 long, 2.56 wide. Abdomen 3.80 long, 2.80 wide. Eye sizes and interdistances: AME 0.14, ALE 0.19, PME 0.16, PLE 0.19; AME-AME 0.06, AME-ALE 0.06, PME-PME 0.10, PME-PLE 0.09. Leg measurements: I 15.12 (4.17, 4.87, 3.20, 2.34); II 13.37 (3.72, 4.25, 3.25, 2.15); III 12.76 (3.44, 3.92, 3.4, 2.00); IV 16.66 (4.68, 5.00, 4.61, 2.37). Chelicerae as in male. Epigyne: atria large, occupying 1/2 of epigynal plate, located anteromedially; hoods distinct, located anterolaterally; spermathecae spiraled; copulatory ducts long and almost horizontally stretched (Fig. 8A–B).

##### Distribution.

Known only from the type locality (Fig. 12).

#### Spiricoelotes xianheensis

Taxon classificationAnimaliaAraneaeAgelenidae

Chen & Li sp. n.

http://zoobank.org/43D32E4A-EEA8-4015-AEF2-8F43A9C3EF79

Figs 9 , 10 , 12

##### Type material.

**Holotype** ♂: China: Jiangxi: Ganzhou City: Chongyi County: Niedu Village, Xianhe Cave, N25°28'40", E114°06'56", elevation: 402 m, 23.IV.2013, Y.F. Luo and J.C. Liu. **Paratypes**: 1♂1♀, same data as holotype.

##### Etymology.

The specific name refers to the type locality; adjective.

##### Diagnosis.

**Figure 9.**
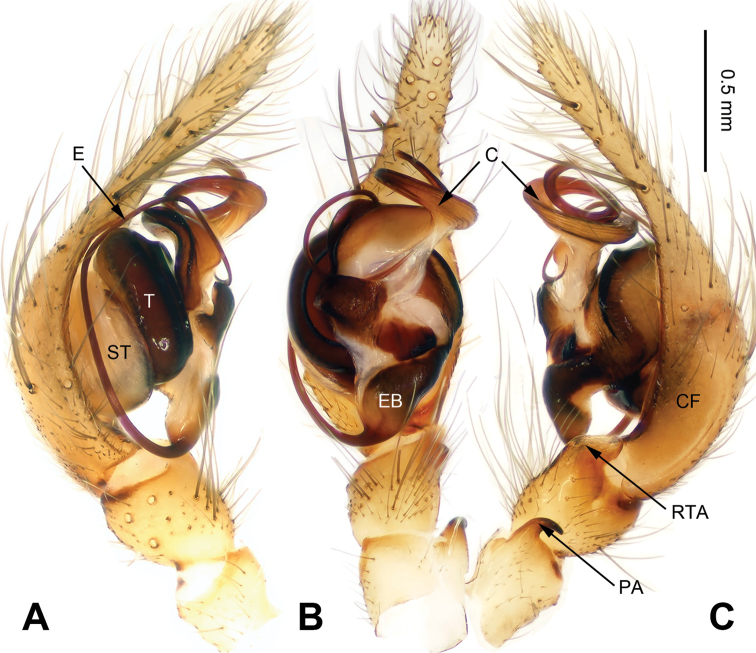
Left palp of *Spiricoelotes xianheensis* sp. n., holotype. **A** Prolateral **B** Ventral **C** Retrolateral. Scale bar equal for **A, B** and **C**.

**Figure 10.**
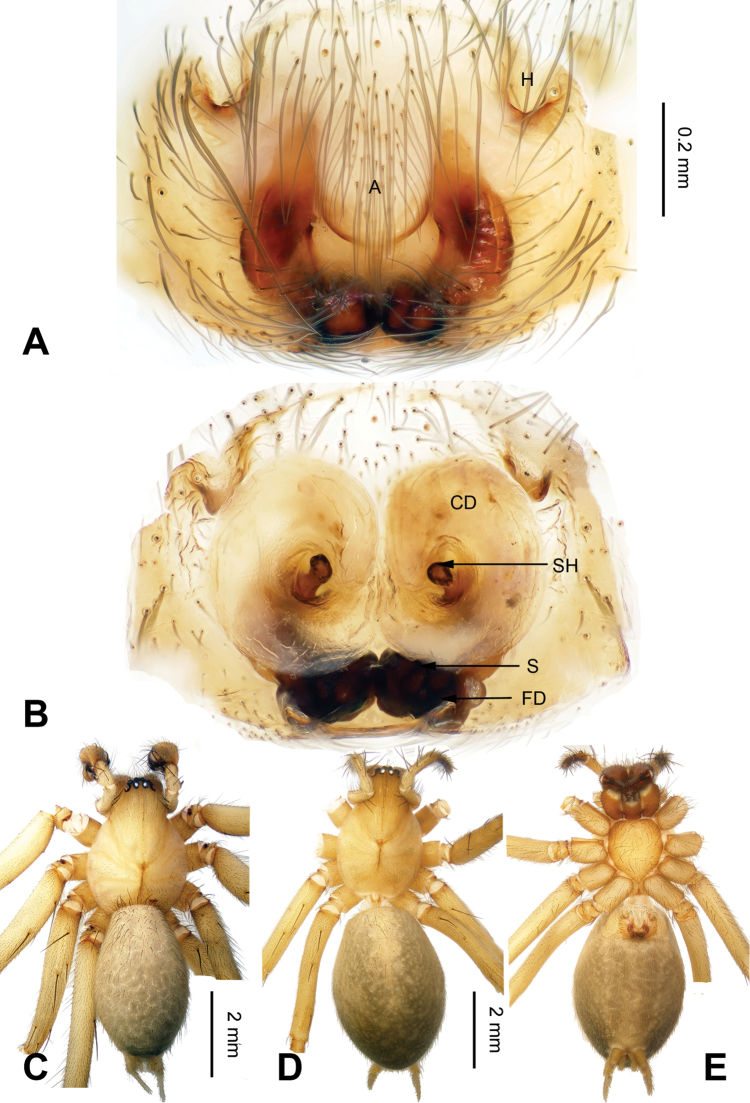
Epigyne and habitus of *Spiricoelotes xianheensis* sp. n., **A** Epigyne, ventral **B** Vulva, dorsal **C** Male habitus, dorsal **D** Female habitus, dorsal **E** Female habitus, ventral. Scale bars equal for **A** and **B**, equal for **D** and **E**.

The male of the new species is similar to *Spiricoelotes anshiensis* sp. n. and *Spiricoelotes nansheensis* sp. n. but can be distinguished from them by the less modified conductor (conductor without outgrowth at base in this species, with one outgrowth in *Spiricoelotes anshiensis* sp. n. and 2 outgrowths in *Spiricoelotes nansheensis* sp. n.) and having more loops in conductor (2 loops in this species, 1 loop in *Spiricoelotes anshiensis* sp. n. and *Spiricoelotes nansheensis* sp. n.) (Fig. 9A–C). The female of the new species has a uniquely shaped epigyne and can be distinguished from all of the other *Spiricoelotes* species by the medially situated and U-shaped atrium and the copulatory ducts that are looped around the spermathecal heads (Fig. 10A–B).

##### Description.

**Male (holotype)**: Total length 6.90. Carapace 3.20 long, 2.25 wide. Abdomen 3.70 long, 2.13 wide. Eye sizes and interdistances: AME 0.13, ALE 0.14, PME 0.15, PLE 0.17; AME-AME 0.05, AME-ALE 0.02, PME-PME 0.08, PME-PLE 0.06. Leg measurements: I 14.76 (3.96, 4.95, 3.55, 2.30); II 14.23 (3.88, 4.80, 3.40, 2.15); III 13.08 (3.60, 3.80, 3.40, 2.28); IV 17.32 (4.50, 5.13, 5.13, 2.56). Chelicerae with 3 promarginal and 4 retromarginal teeth. Palp: patellar apophysis shorter than patellar width, strongly curved; RTA with blunt tip, extending beyond tibia; LTA short, about 1/4 length of RTA; cymbial furrow long, approximately 1/3 length of cymbium; conductor long, anteriorly extending, with looped apex; embolus beginning at 6 o’clock position; base of embolus rather small, ovoid, almost vertically directed (Fig. 9A–C).

**Female (one of paratypes)**: Total length 7.87. Carapace 3.32 long, 2.28 wide. Abdomen 4.55 long, 3.00 wide. Eye sizes and interdistances: AME 0.13, ALE 0.14, PME 0.13, PLE 0.12; AME-AME 0.05, AME-ALE 0.04, PME-PME 0.09, PME-PLE 0.11. Leg measurements: I 14.12 (3.60, 4.60, 3.68, 2.24); II 12.32 (3.40, 3.92, 3.00, 2.00); III 12.38 (3.32, 3.76, 3.20, 2.10); IV 15.32 (4.17, 4.68, 4.17, 2.30). Chelicerae as in male. Epigyne: atrium small, situated medially, U-shaped; hoods distinct, located anterolaterally; spermathecae long, convoluted; spermathecal heads small; copulatory ducts broad, looped around spermathecal heads (Fig. 10A–B).

##### Distribution.

Known only from the type locality (Fig. 12).

#### Spiricoelotes xiongxinensis

Taxon classificationAnimaliaAraneaeAgelenidae

Chen & Li sp. n.

http://zoobank.org/24ED870E-54BB-4D51-8B25-C6E30552F146

Figs 11 , 12

##### Type material.

**Holotype** ♀: China: Jiangxi: Pingxiang City: Shangli County: Futian Town, Zhanshan Village, Xiongxin Cave, N27°44'08", E113°49'10", elevation: 115 m, 9.V.2013, Y.F. Luo and J.C. Liu.

##### Etymology.

The specific name refers to the type locality; adjective.

##### Diagnosis.

**Figure 11.**
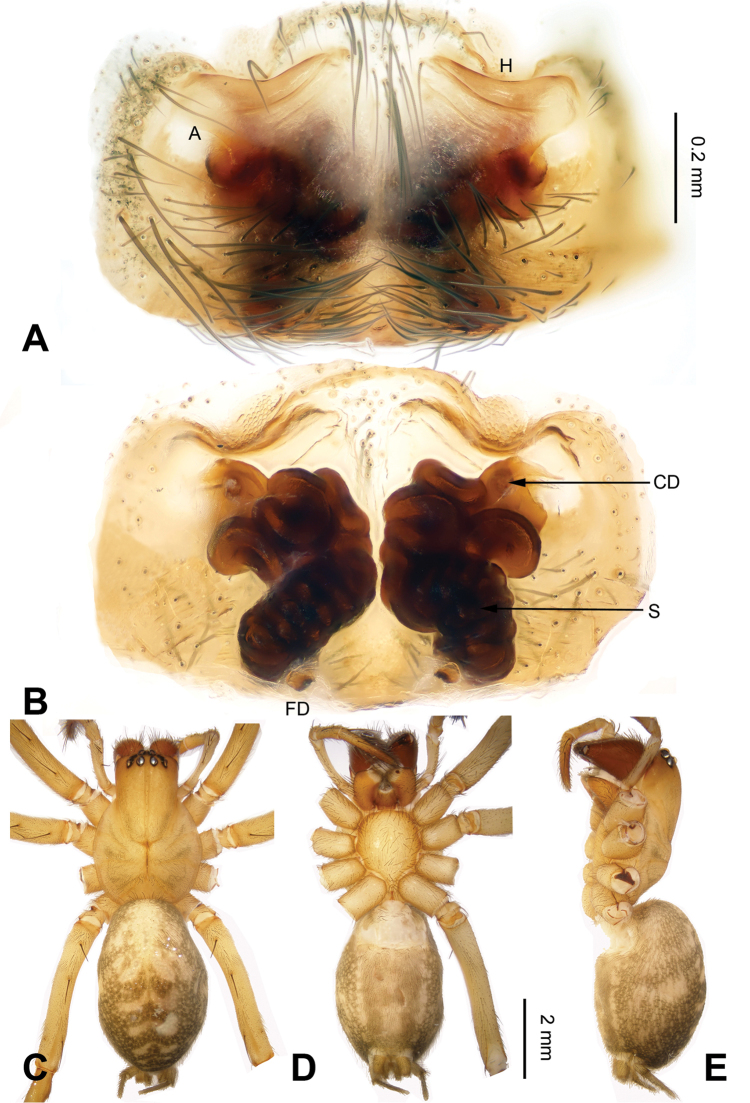
Epigyne and habitus of *Spiricoelotes xiongxinensis* sp. n., holotype. **A** Epigyne, ventral **B** Vulva, dorsal **C** Female habitus, dorsal **D** Female habitus, ventral **E** Female habitus, lateral. Scale bars: equal for **A** and **B**, equal for **C, D** and **E**.

The female of the new species has a uniquely shaped epigyne and can be easily distinguished from all of the other *Spiricoelotes* species by the small and anteriorly situated epigynal atria, the broad epigynal hoods, the long and convoluted spermathecae and by the short copulatory ducts (Fig. 11A–B).

##### Description.

**Female (holotype)**: Total length 8.08. Carapace 3.85 long, 2.69 wide. Abdomen 4.23 long, 2.69 wide. Eye sizes and interdistances: AME 0.19, ALE 0.20, PME 0.19, PLE 0.18; AME-AME 0.11, AME-ALE 0.04, PME-PME 0.13, PME-PLE 0.14. Leg measurements: I 13.76 (3.72, 4.56, 3.36, 2.12); II 12.08 (3.44, 4.08, 3.00, 1.56); III 11.68 (3.20, 3.88, 3.04, 1.56); IV 15.66 (4.20, 4.75, 4.5, 2.21). Chelicerae with five promarginal and five retromarginal teeth. Epigyne: atria small, located anterolaterally, distantly separated by septum; hoods distinct, located at anterior atrial margin; spermathecae long, convoluted; copulatory ducts broad and short, located anteriorly and horizontally directed (Fig. 11A–B).

##### Distribution.

**Figure 12.**
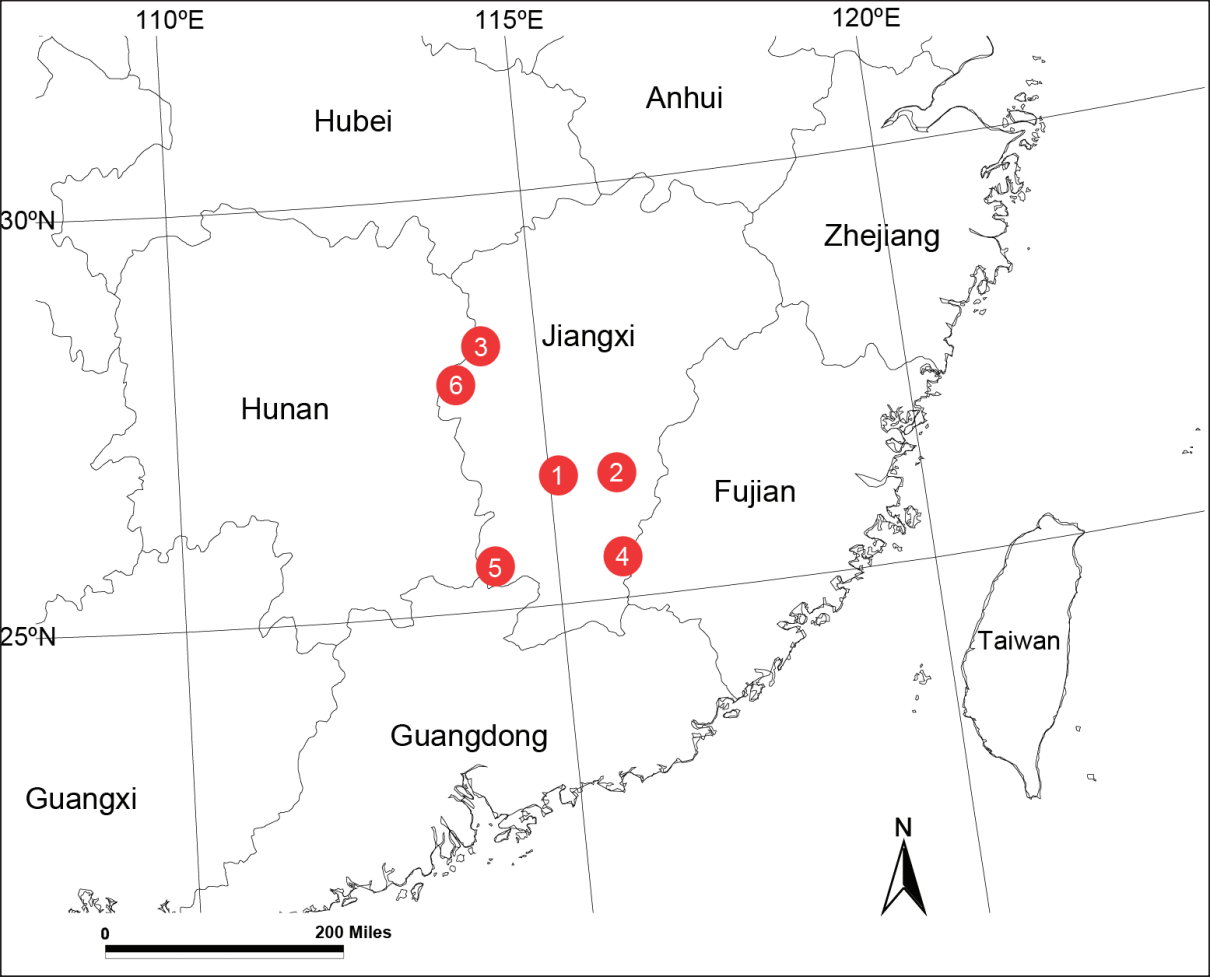
Localities of new *Spiricoelotes* species from China. **1** *Spiricoelotes anshiensis* sp. n. **2** *Spiricoelotes chufengensis* sp. n. **3** *Spiricoelotes nansheensis* sp. n. **4** *Spiricoelotes taipingensis* sp. n. **5** *Spiricoelotes xianheensis* sp. n. **6** *Spiricoelotes xiongxinensis* sp. n.

Known only from the type locality (Fig. 12).

## Discussion

Currently nine species are known in the genus. Seven are known from both sexes and two from females only. The conductors of the five newly described species differ to a certain extent from the two previously known species. The tip of the conductor in *Spiricoelotes urumensis* and *Spiricoelotes zonatus* is spiralled, whereas in the newly described species, it has a looped apex or is unmodified, but never spiralled. All new species are assigned to this genus because they share a combination of characters with *Spiricoelotes zonatus*, ﻿the type species of *Spiricoelotes* that are lacking in other Coelotinae: strongly curved patellar apophysis, conductor anteriorly extending, median apophysis and dorsal apophysis of conductor absent (figs 1A–C, 3A–C, 5A–C, 7A–C, 9A–C; Wang 2002: figs 366–368).

## Supplementary Material

XML Treatment for Spiricoelotes

XML Treatment for Spiricoelotes anshiensis

XML Treatment for Spiricoelotes chufengensis

XML Treatment for Spiricoelotes nansheensis

XML Treatment for Spiricoelotes taipingensis

XML Treatment for Spiricoelotes xianheensis

XML Treatment for Spiricoelotes xiongxinensis
